# A Focused Review on Commercially Available Automated Systems for Antibiotic Susceptibility Testing

**DOI:** 10.1155/jotm/9694354

**Published:** 2025-11-25

**Authors:** Qumars Ghavami, Haider Abd Ulhai Nasser, Seyedeh Shadi Vaziri, Majid Taati Moghadam

**Affiliations:** ^1^Department of Microbiology, Faculty of Veterinary Science, Bu-Ali Sina University, Hamedan, Iran; ^2^Department of Mathematics, Faculty of Basic Education, Babylon University, Babil, Iraq; ^3^Department of Microbiology, Faculty of Basic Silences, Shahrekord Branch, Islamic Azad University, Shahrekord, Iran; ^4^Department of Microbiology, Virology and Microbial Toxins, School of Medicine, Guilan University of Medical Sciences, Rasht, Iran

**Keywords:** antibiotic resistance, antibiotic susceptibility testing, automated systems

## Abstract

The battle against antibiotic resistance demands innovative solutions for efficient and accurate antibiotic susceptibility testing (AST). Traditional methods, though reliable, are often time-consuming and labor-intensive, limiting their ability to provide timely results in clinical settings. In response, automated systems have emerged as a revolutionary tool, offering rapid, precise, and comprehensive AST processes. The purpose of this review article was to provide a comprehensive guide on automated methods for AST, exploring their principles, advantages over traditional techniques, and implications for clinical practice. By examining recent advancements in this field, we highlight how these innovative approaches can revolutionize our ability to detect antibiotic resistance swiftly and accurately, ultimately improving patient outcomes and combating the growing threat of antimicrobial resistance. The findings from the collected studies indicate that employing various automated techniques for assessing antibiotic resistance facilitates the identification of resistance and allows for precise detection of different pathogens from infectious samples. These automated methods have the potential to decrease hospital stay duration and future treatment costs. Moreover, by accurately detecting resistance more quickly, they can lead to more targeted treatments, ultimately reducing patient mortality rates.

## 1. Introduction

**Figure 1:**
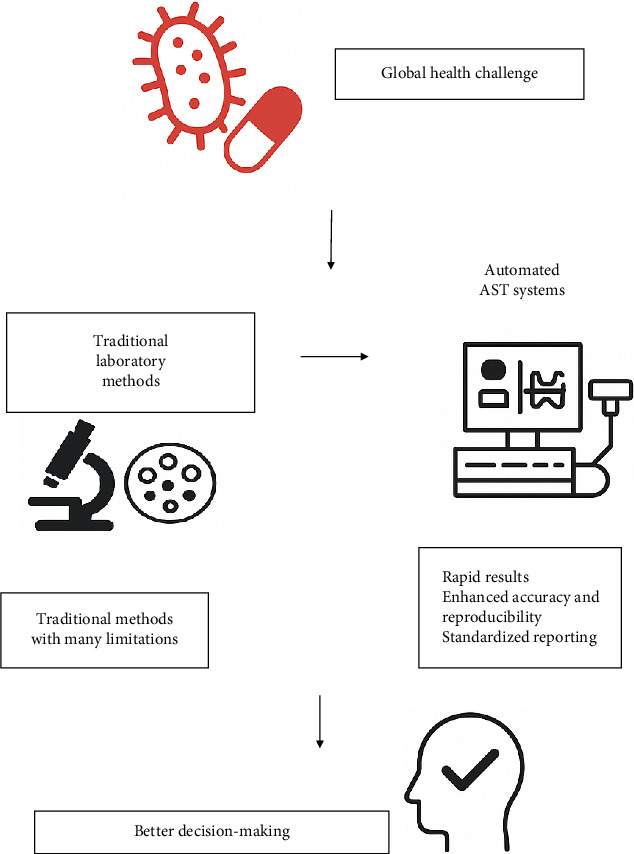
The schematic illustrates the transition from traditional laboratory AST methods—characterized by time-consuming processes, limited sensitivity and specificity, resource constraints, and interpretative variability—to automated AST systems offering rapid results, enhanced accuracy and reproducibility, and standardized reporting. This advancement supports improved decision-making strategies in tackling global health challenges related to infectious diseases.

Antibiotic resistance has emerged as one of the most significant global health challenges of the twenty-first century. The history of antibiotic resistance dates back to the early days of antibiotic discovery, with the first modern antibiotics, such as penicillin, introduced in the 1940s. Initially celebrated as miracle drugs that could effectively treat bacterial infections, these antibiotics soon faced the inevitable challenge of resistance. As early as 1940, shortly after penicillin's introduction, researchers identified bacterial strains capable of inactivating penicillin through enzymatic degradation [1–3]. This phenomenon has persisted and evolved, leading to a complex landscape of antimicrobial resistance (AMR) that now threatens to undermine decades of medical progress [4–6]. By using antibiotic susceptibility testing (AST) to inform treatment decisions, unnecessary use of broad-spectrum antibiotics can be minimized. This targeted approach helps to preserve the effectiveness of existing antibiotics and slows down the emergence of antibiotic-resistant strains of bacteria, a growing public health concern [7, 8]. In response to traditional method challenges, automated methods for detecting antibiotic resistance have been developed and are gaining traction in clinical laboratories. Automated AST systems utilize advanced technologies, such as microfluidics and robotics, to streamline the testing process. These systems can rapidly analyze multiple samples simultaneously, significantly reducing turnaround times from days to hours [9]. Furthermore, automated methods can detect a broader range of antibiotic resistances due to their ability to perform complex analyses that traditional methods may overlook. The advantages of automated AST systems extend beyond speed; they also enhance accuracy and reproducibility. Automated systems minimize human error associated with manual techniques and provide standardized results that facilitate better decision-making in clinical settings (Figure 1). Additionally, these systems can integrate with laboratory information management systems (LIMS), allowing for seamless data management and reporting [10]. The purpose of this review article was to provide a comprehensive guide on automated methods for AST, exploring their principles, advantages over traditional techniques, and implications for clinical practice. By examining recent advancements in this field, we highlight how these innovative approaches can revolutionize our ability to detect antibiotic resistance swiftly and accurately, ultimately improving patient outcomes and combating the growing threat of AMR.

## 2. Challenges of AMR and Traditional Methods

**Figure 2:**
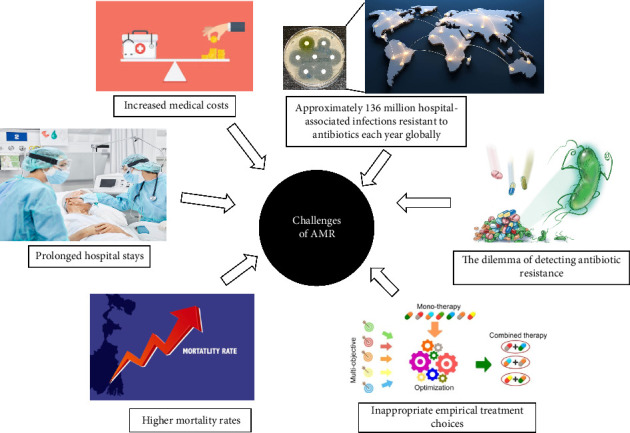
The significant clinical challenges of dealing with antibiotic resistance.

The challenges posed by antibiotic resistance in clinical settings are multifaceted (Figure 2). Infections caused by resistant bacteria often result in prolonged hospital stays, increased medical costs, and higher mortality rates. For instance, the Centers for Disease Control and Prevention (CDC) estimates that at least 2.8 million people in the United States are infected with antibiotic-resistant bacteria each year, resulting in over 35,000 deaths [4, 11, 12]. The economic burden on healthcare systems is staggering; it is estimated that antibiotic-resistant infections cost the U.S. healthcare system approximately $20 billion annually in direct medical costs and up to $35 billion in lost productivity [13]. Bloodstream infections caused by antibiotic-resistant bacteria lead to increased mortality rates in low- and middle-income countries. The excess mortality associated with antibiotic-resistant bacterial infections is substantial, though specific rates can vary widely depending on the region and type of infection. The economic burden of antibiotic-resistant bacterial infections includes increased direct medical costs due to longer hospital stays and more intensive care requirements [14]. Children hospitalized with pneumonia who had bacteremia from antibiotic-resistant strains exhibited a mortality rate of 29%, significantly higher than the 7% mortality rate for those without bacteremia. This stark contrast underscores the critical impact of antibiotic resistance on patient outcomes, particularly among vulnerable populations [15]. Overall, it is estimated that there are approximately 136 million hospital-associated infections resistant to antibiotics each year globally, with a significant burden in countries, such as China, Pakistan, and India. Middle-income countries bear the highest annual incidence of such infections [16, 17]. Beyond direct healthcare costs, the economic impact also includes productivity losses from years of potential life lost due to premature deaths caused by these antibiotic-resistant infections. This indirect cost is particularly pronounced in low- and middle-income countries where the workforce is often younger and more affected by these health issues [14, 18]. This financial strain is compounded by the fact that many commonly used antibiotics are becoming increasingly ineffective against prevalent pathogens. The dilemma of detecting antibiotic resistance adds another layer of complexity to this issue. AST helps clinicians select the most effective antibiotics for treating bacterial infections, which is essential for improving patient outcomes and reducing the risk of treatment failure. By identifying the specific resistance patterns of bacteria, healthcare providers can tailor antibiotic therapy to the individual patient, ensuring that the chosen antibiotic will effectively combat the infection [7, 19].

**Figure 3:**
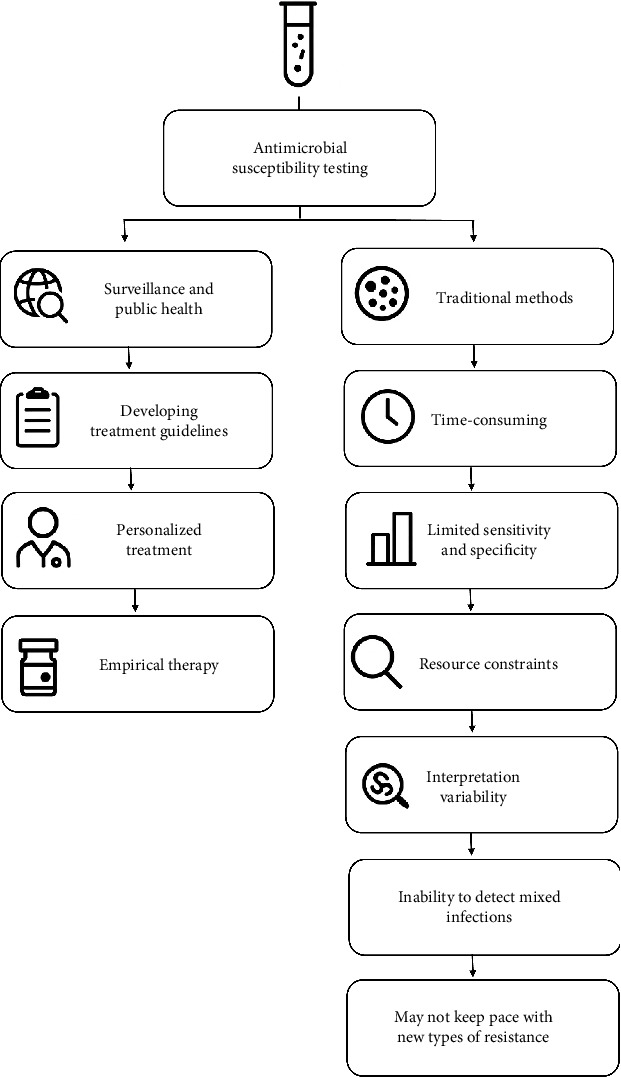
This figure illustrates the dual role and challenges of antimicrobial susceptibility testing, highlighting its impact on guiding surveillance, public health, treatment guidelines, personalized therapies, and empirical therapy (left branch), while simultaneously showcasing key limitations inherent to traditional testing methods (right branch), such as time consumption, limited sensitivity, resource constraints, interpretation variability, difficulty detecting mixed infections, and the inability to keep pace with emerging antimicrobial resistances. This holistic visual summary underscores both the clinical value and the diagnostic obstacles faced in implementing and advancing antimicrobial stewardship programs.

Data generated from AST can contribute to surveillance efforts and inform public health policies. Understanding local resistance patterns assists in developing empirical treatment guidelines and can influence antibiotic stewardship programs aimed at optimizing antibiotic use across healthcare settings [7, 19]. AST allows for personalized treatment plans based on individual bacterial profiles, leading to better clinical outcomes. Selecting the right antibiotic from the start can lead to quicker recovery times for patients, reducing hospital stays and associated healthcare costs [19, 20]. In cases where immediate treatment is necessary, AST data can guide empirical therapy choices, ensuring they are more likely to be effective against prevalent local pathogens [21]. By avoiding ineffective antibiotics, AST reduces the risk of adverse drug reactions and complications associated with inappropriate treatment [19, 20, 22]. Many countries still use traditional methods to perform AST. Traditional methods for identifying antibiotic resistance in the laboratory, while foundational, face several significant challenges, including time-consuming processes that can take 24 to 48 h to yield results (this timeframe applies mostly to fast-growing bacteria commonly responsible for routine infections in hospitals and communities. However, it is not suitable for slow-growing bacteria, such as mycobacteria, for which the timeframe is significantly longer—often exceeding 20 days), limited sensitivity and specificity (may not detect all resistance mechanisms), laboratory resource constraints (may be inadequate infrastructure or trained personnel to perform comprehensive AST), variability in interpretation based on the methodology used and the standards applied, traditional methods, which may not keep pace with new types of resistance or emerging resistance mechanisms, inability to detect mixed infections, and cost and accessibility in low-resource settings where access to necessary reagents and equipment is limited (Figure 3) [14, 23–29].

**Figure 4:**
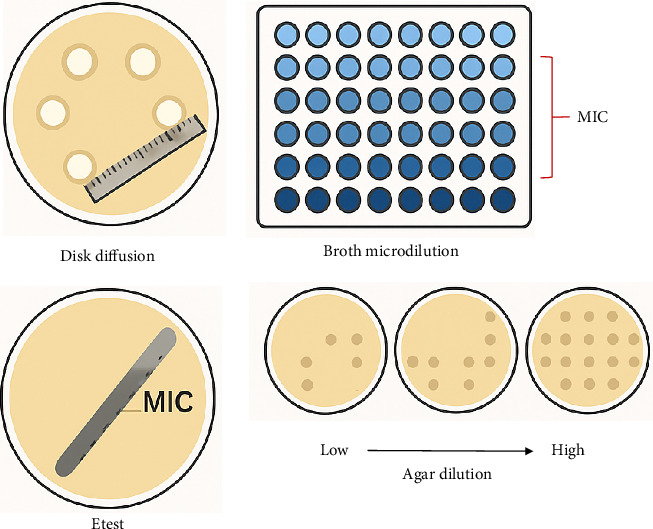
Schematic illustration of common laboratory methods for determining antimicrobial susceptibility.

Several traditional methods are commonly used for AST: (1) Disk Diffusion Method: This is one of the most widely used methods where antibiotic-impregnated paper disks are placed on an agar plate inoculated with the bacteria in question. After incubation, the zones of inhibition around each disk are measured to determine susceptibility or resistance based on established standards (Clinical Laboratory Standards Institute—CLSI guidelines) [30, 31]. (2) Broth Microdilution: This method involves diluting antibiotics in a liquid growth medium containing bacterium. The lowest concentration of antibiotic that inhibits visible growth (minimum inhibitory concentration or MIC) is determined. This method provides quantitative results regarding bacterial susceptibility [7, 22]. (3) Etest (Epsilometer Test): This combines aspects of both disk diffusion and broth dilution methods. A strip containing a gradient of antibiotic concentrations is placed on an agar plate inoculated with bacteria. The MIC is determined by reading where bacterial growth intersects with the strip [31, 32]. (4) Agar Dilution Method: In this method, different concentrations of antibiotics are incorporated into agar plates. Bacteria are then inoculated onto these plates, and growth is assessed to determine susceptibility (Figure 4) [33]. Traditional methods for determining antibiotic susceptibility often rely on culture-based techniques that can take several days to yield results. This delay can lead to inappropriate empirical treatment choices, where broad-spectrum antibiotics are prescribed without knowing the specific resistance profile of the infecting organism. Consequently, patients may receive ineffective treatments while waiting for laboratory results, which can worsen their condition and contribute to further resistance development. Consequently, there is an urgent need for more efficient and reliable testing methods [34, 35]. Therefore, the emergence of automated AST techniques has addressed many of the challenges associated with traditional methods. It is essential to update these methods to meet clinical and patient needs.

## 3. Commercially Available Automated Detection Systems in Clinical Setting

Traditional blood culture techniques are considered the benchmark for diagnosing sepsis and guiding patient treatment. However, obtaining antibiotic susceptibility results typically requires 24 to 48 h after a blood culture tests positive. Over the last 10 years, advancements have introduced highly automated and sensitive blood culture systems. Additionally, rapid bacterial identification and molecular susceptibility assays conducted simultaneously have further shortened turnaround times. Nevertheless, comprehensive and dependable AST still depends on full-panel phenotypic methods. In clinical microbiology laboratories, phenotypic AST is generally performed with automated instruments, which rely on preparing a standardized inoculum from isolated colonies grown overnight on solid media, following the manufacturer's protocols [36]. The European Committee on Antimicrobial Susceptibility Testing (EUCAST) has validated a rapid AST (RAST) procedure that measures inhibition zones on Mueller–Hinton agar plates after 4, 6, and 8 h of incubation. Meanwhile, the French Microbiology Society's antibiotics committee (CA-SFM) assessed a modified EUCAST RAST protocol that omits subculturing by directly inoculating positive blood culture broth onto Mueller–Hinton agar plates, enabling results to be obtained one day sooner compared to traditional methods. Although conventional AST remains the standard, studies of RAST paired with the VITEK 2 system show it can deliver dependable results a day earlier, and the approach is simple, fast, and cost-effective. Multicenter evaluations of platforms, such as VITEK 2 and BD Phoenix, have demonstrated high levels of essential agreement (EA) and categorical agreement (CA)—often exceeding 95%—with reference broth microdilution methods, supporting their clinical reliability [36–38]. These RAST strategies have a promising role in enhancing clinical decision-making and in curbing the rise of antimicrobial-resistant bacteria by minimizing inappropriate or inadequate antibiotic use. Such misuse is frequently linked to longer hospital stays, multidrug-resistant infections, higher mortality rates, and increased healthcare expenses. Omitting centrifugation and lysis steps could further streamline workflows and reduce turnaround times for processing positive blood culture broths directly on systems, such as VITEK 2, in routine clinical microbiology settings [36–38].

The automated AST systems have significantly transformed the landscape of clinical microbiology, enhancing the speed and accuracy of diagnosing infections and guiding effective treatment (Table 1). Traditional methods, such as broth microdilution and disk diffusion, have been the mainstay for AST; however, they are often time-consuming and labor-intensive. Automated systems have emerged to streamline these processes, allowing for rapid identification and susceptibility testing of pathogens directly from clinical samples [50–53]. Automated AST systems utilize advanced technologies, including mass spectrometry and microfluidics, which facilitate high-throughput testing and provide real-time results. These innovations not only reduce the turnaround time for test results but also improve the reliability of susceptibility data by minimizing human error associated with manual techniques [50, 52, 54, 55]. Furthermore, the integration of automated systems into clinical workflows has been shown to enhance patient outcomes by enabling timely administration of appropriate antibiotics, particularly in critical care settings where prompt treatment is crucial [50, 51, 53]. The ability to quickly identify resistant strains not only aids clinicians in selecting appropriate therapies but also supports public health initiatives aimed at monitoring and controlling AMR trends. Despite their promise, automated methods require rigorous validation before widespread implementation in clinical practice. Validation ensures that these new technologies produce reliable results comparable to traditional methods while also demonstrating their effectiveness across diverse bacterial species and resistance mechanisms [56]. Despite these advancements, challenges remain. The transition to automated systems requires substantial investment in technology and training for laboratory personnel. Additionally, while many automated methods are already commercialized, some are still in developmental stages or limited to specific pathogens [50, 52, 57]. Overall, the ongoing evolution of automated AST systems represents a pivotal step toward combating antibiotic resistance and improving infectious disease management in healthcare settings. In this section, we will discuss the most significant automated AST systems.

### 3.1. VITEK 2 (bioMérieux)

VITEK 2 (bioMérieux) is an automated system widely used in clinical microbiology for the identification of microorganisms and the determination of their antibiotic susceptibility. This system streamlines the testing process, providing rapid and reliable results that are crucial for effective patient management. The VITEK 2 system employs a combination of microbial identification and AST based on the principles of colorimetric detection and optical density measurement. The system uses cards that contain multiple wells, each with a different antibiotic concentration. The system identifies bacteria based on their metabolic activity, which is detected by color changes in the wells containing specific substrates. The growth or inhibition of bacteria in the presence of antibiotics is monitored. The MIC is determined by observing the lowest concentration of an antibiotic that prevents visible growth [58–61]. The steps involved in using VITEK 2 for AST include the following: (1) Sample Collection: Collect clinical specimens (e.g., blood, urine, and swabs) from patients suspected of infection. (2) Isolation and Identification: Inoculate the specimen onto appropriate culture media to isolate the pathogen. After incubation, isolated colonies are selected for further testing. This ensures that only one type of microorganism is being tested at a time. (3) Preparation of Inoculum: Prepare a standardized bacterial suspension (usually 0.5 McFarland standard) from isolated colonies to ensure consistent inoculum density. (4) Loading the VITEK Card: Inoculate a VITEK AST card with the prepared bacterial suspension. Each card contains wells with different antibiotics at varying concentrations. Perform preliminary identification using biochemical tests or directly using VITEK 2 identification cards based on metabolic activity detected in the wells of the card. Simultaneously, the system monitors bacterial growth in the presence of different antibiotics to determine susceptibility [62–65]. (5) Incubation: Place the inoculated card into the VITEK 2 instrument, which incubates the card at controlled temperatures and monitors bacterial growth. (6) Monitoring and Data Collection: The system continuously measures changes in color and turbidity in each well, indicating bacterial growth or inhibition. (7) Result Interpretation: After incubation, the VITEK 2 analyzes the data and generates an automated report. Results are classified as follows: Susceptible (S): Bacteria are inhibited by standard concentrations of antibiotics; Intermediate (I): Bacteria may be inhibited by higher concentrations or may show variable responses; and Resistant (R): Bacteria grow despite the presence of antibiotics. (8) Reporting: Results are printed or electronically transmitted to clinicians, aiding in selecting appropriate antibiotic therapy [59, 63, 64, 66]. Researchers conducted a multicenter evaluation of RAST using VITEK 2 directly from positive blood cultures. The study focused on patient samples with monomicrobial infections caused by Gram-negative rods or Gram-positive cocci in clusters, analyzing results from Day 0 and comparing them to Day 1 AST results across seven French laboratories. The overall CA and EA rates were notably high at 98.4% and 96.7%, respectively. For Enterobacterales and Staphylococci, the rates of very major discrepancies and major discrepancies met the NF EN ISO 20776-2 (2007) criteria for drugs relevant to sepsis treatment. However, significant very major discrepancies were observed for certain antibiotics: amoxicillin–clavulanate (4.9%), piperacillin–tazobactam (7.5%), meropenem (33%), and gentamicin for Staphylococci (4.6%). Overall, direct AST from positive blood culture broths using VITEK 2 proved reliable and efficient for Enterobacterales and Staphylococci, potentially enhancing antimicrobial stewardship [36]. Dwivedi et al. conducted an evaluation of the VITEK 2 system for meropenem–vaborbactam susceptibility testing in Enterobacterales and *Pseudomonas aeruginosa*. Among 449 Enterobacterales isolates assessed using FDA/CLSI breakpoints, the results showed a high level of accuracy: 98.2% EA and 98.7% CA, with no instances of very major errors (VMEs) or major errors (MEs). When applying EUCAST breakpoints for analysis across both Enterobacterales and *P. aeruginosa*, VITEK 2 demonstrated strong performance relative to reference broth microdilution methods: Overall EA was at 97.3%, and CA reached up to 99.2%, while VMEs were observed in about one-fifth of cases before error resolution. Specifically for *P. aeruginosa*, results included an EA rate of approximately two-thirds below perfect alignment but maintained zero instances of very major discrepancies; after addressing discrepancies through further review, these figures improved marginally. For Enterobacterales alone under EUCAST guidelines, performance metrics indicated a high degree of reliability with nearly all isolates correctly categorized as susceptible or resistant without significant error—though some minor discrepancies existed initially which were largely resolved upon reevaluation. These positive findings supporting VITEK's accuracy in automated susceptibility testing compared against manual methods, such as broth microdilution tests, are used traditionally as benchmarks in clinical microbiology laboratories worldwide today [38]. A comprehensive analysis involving cultural traits, automated biochemical testing, and PCR-based invA gene detection led to the identification of 134 *Salmonella* isolates. The susceptibility of these isolates to antibiotics was assessed using the VITEK 2 system. Utilizing both VITEK 2 ID-GN cards and invA gene detection methods revealed seven distinct *Salmonella* serotypes. The most prevalent serovars included *Salmonella typhimurium, Salmonella paratyphi, Salmonella enteritidis, Salmonella enterica*, and *Salmonella typhi.* In contrast, fewer instances of *Salmonella gallinarum* and *Salmonella arizonae* were observed. Of the total of 136 isolates analyzed at the species level, an impressive accuracy rate of approximately 98.5% was achieved in identifying them correctly as belonging to specific species—134 out of every possible case being accurately classified. Further categorization showed that about half fell into higher quality categories: nearly half at excellent levels (47%), followed by very good at almost a third (29%), then smaller percentages categorized as good or acceptable—around one-tenth for each category, respectively. Seven serotypes demonstrated VMEs in their identification profiles during testing. MEs occurred in 15 cases, while minor errors affected 67 organisms overall. Overall EA was calculated at approximately 98.86%, with a CA rate around 95.95%. The most common multidrug resistance pattern observed among these serovars involved resistance to ampicillin (AMP), chloramphenicol, sulfonamide, and trimethoprim compounds collectively [67].

### 3.2. Phoenix (BD)

The Phoenix system (BD) is an automated microbiology platform designed for the identification of microorganisms and the determination of their antibiotic susceptibility. It is widely used in clinical laboratories to streamline the process of diagnosing infections and guiding appropriate antibiotic therapy [68, 69]. Principles of the Phoenix system in AST contains the Phoenix system operates on the principles of microbial identification and AST through a combination of biochemical reactions and optical detection methods [70]. The system uses a series of biochemical tests to identify bacteria based on their metabolic activities, such as substrate utilization and enzyme production [68, 71]. The system assesses bacterial growth in the presence of various antibiotics, determining the MIC for each antibiotic tested [69, 72]. This is achieved through colorimetric changes that indicate bacterial growth or inhibition [73, 74]. The steps involved in using the Phoenix system for AST contain the following: (1) Sample Collection: Clinical specimens (e.g., blood, urine, and swabs) are collected from patients suspected of having bacterial infections [69, 75]. (2) Culture: The collected samples are inoculated onto appropriate culture media to isolate bacterial colonies. The BD Phoenix system allows for the direct inoculation of samples, such as blood culture broths, for AST without the need for prior subculturing. This method has been shown to provide rapid results, significantly reducing turnaround time for susceptibility testing [76, 77]. (3) Isolation of Bacteria: After incubation, isolated colonies are selected for further testing. This ensures that only one type of microorganism is being analyzed [69, 75]. (4) Preparation of Inoculum: A standardized bacterial suspension is prepared from the isolated colonies, typically matching a 0.5 McFarland standard, which corresponds to approximately 1.5 × 10^8^ CFU/mL [69]. (5) Inoculation into Phoenix Panels: The prepared suspension is inoculated into a Phoenix AST panel, which contains wells with various antibiotics at different concentrations [69, 75, 78]. (6) Incubation: The inoculated panel is placed in the Phoenix instrument, which incubates it under controlled conditions while continuously monitoring changes in color and turbidity in each well [76]. (7) Monitoring and Data Collection: The system uses optical sensors to detect metabolic activity and growth patterns in real-time. It assesses whether bacteria can grow in the presence of antibiotics by monitoring color changes in the wells [76, 78]. (8) Result Interpretation: After incubation, the Phoenix system analyzes the data and generates a report indicating whether each antibiotic tested is susceptible, intermediate, or resistant based on established breakpoints [69, 75, 79]. A research study examined the effectiveness of the Phoenix automated microbiology system in identifying and conducting AST on staphylococci and enterococci isolates collected from patients in a tertiary-care hospital. The study analyzed a total of 424 isolates, which included 90 enterococci, 232 *Staphylococcus aureus* isolates (with 14 vancomycin-intermediate strains), and 102 other staphylococcal species. The study used conventional biochemical methods and cell wall fatty acid analysis with the Sherlock microbial identification system as reference tools for comparison. Agar dilution served as the reference method for AST. The overall concordance rates for genus and species identification were 99.7% and 99.3%, respectively. Both *S. aureus* and enterococci were accurately identified using the Phoenix system. For the non-*S. aureus* staphylococci, there was a 98% agreement rate in identifying 16 different species. The AST results were categorized by organism type. For *S. aureus*, the CA was 98.2%, and the EA was 98.8%. Overall, the Phoenix system performed comparably to traditional methods for identifying and conducting AST on these microorganisms [37]. The effectiveness of the BD Phoenix CPO Detect (PCD) assay in identifying and categorizing carbapenemases was evaluated using a set of 194 carbapenem-resistant clinical isolates, comprising Enterobacterales (*n* = 65) and nonfermentative Gram-negative rods (*n* = 129). The performance assessment revealed that the PCD assay demonstrated an overall sensitivity of 98.29% but a specificity of 17.95% for detecting carbapenemase production. In terms of classifying carbapenemases into classes A, B, and D, the PCD correctly categorized 79.17% of Enterobacterales and 67.16% of nonfermentative Gram-negative rods. The PCD assay is a dependable method for detecting carbapenem resistance while enabling simultaneous analysis of carbapenemase production. However, despite its high sensitivity, its low specificity in detecting carbapenemases combined with potential misclassification necessitates confirmation through alternative methods—particularly crucial when dealing with nonfermentative Gram-negative bacteria [80]. To evaluate the effectiveness of Etest, VITEK 2, and BD Phoenix in determining the susceptibility of *Streptococcus pneumoniae* to penicillin, AMP, and cefotaxime, a comprehensive assessment was conducted. The results showed that all methods achieved an EA of at least 90% when compared to broth microdilution—except for Etest using benzylpenicillin (BEN) on Oxoid plates (58.3%) and AMP tests on both Oxoid (65.8%) and BD BBL plates (84.2%). CA for penicillin reached or exceeded 90% only with VITEK 2; other methods had CA rates ranging from 74% to 84%. For AMP, none of the methods achieved a CA above 90%, with results spanning from approximately 75.8% to about 88.3%. Cefotaxime testing yielded CA rates between about 87% and just under or at exactly 90%, except for Etest on Oxoid plates which scored lower at around 79%. The study suggests that VITEK 2 and BD Phoenix are dependable tools for obtaining accurate susceptibility data regarding these antibiotics in pneumococcal infections. However, employing Etest BEN or AMP on Oxoid plates poses a risk of underestimating MIC values; thus, such results should be interpreted cautiously—especially if they fall one or two doubling dilutions below clinical breakpoints [81]. In a study by Jayol et al., the effectiveness of the BD Phoenix system, the Rapid Polymyxin NP test, and the BMD method was assessed for identifying colistin resistance in *Enterobacteriaceae*, focusing on strains with MCR-1 and MCR-2. When compared to the BMD reference method, the BD Phoenix system missed detecting ten colistin-resistant isolates, including one each of *Escherichia coli* and *Klebsiella pneumoniae*, seven *Enterobacter* species, and one *S. enterica*. Similarly, the Rapid Polymyxin NP test also failed to identify a single *E. coli* isolate. Both the BD Phoenix system and the Rapid Polymyxin NP test successfully identified the 16 isolates of *E. coli, K. pneumoniae*, and *S. enterica* that carried plasmid-encoded *mcr*-1 and *mcr*-2 genes. While both the BD Phoenix system and the Rapid Polymyxin NP test are dependable for detecting colistin resistance linked to *mcr*-1 and *mcr*-2, the BD Phoenix system showed a notable rate of false susceptibility, suggesting that its results should be validated using the BMD method. In contrast, the Rapid Polymyxin NP test demonstrated strong agreement with the BMD method, providing results quickly, typically within a two-hour timeframe [82].

### 3.3. MicroScan (Beckman Coulter)

MicroScan is an automated system developed by Beckman Coulter for AST. It employs broth microdilution techniques to determine the MICs of various antibiotics against specific bacterial isolates. This system is crucial for guiding effective treatment options, especially in the context of increasing antibiotic resistance. The MicroScan system operates on the principles of broth microdilution, where serial dilutions of antibiotics are prepared in a liquid growth medium [39, 83]. MicroScan is not used to detect microorganisms directly but is an essential tool in the antimicrobial susceptibility testing process after microorganisms have been isolated and identified. MicroScan can be paired with identification panels that help in the preliminary identification of bacteria before susceptibility testing. This process typically involves other methods or systems to confirm the presence of specific microorganisms. The key principles include MIC (the lowest concentration of an antibiotic that prevents visible growth of a bacterium after a specified incubation period), automated reading (the system automatically reads the results after incubation, providing accurate and objective assessments of bacterial growth), and standardized protocols (the system follows guidelines from organizations, such as the CLSI to ensure reliability and reproducibility in results) [78, 84, 85]. The steps involved in using MicroScan for AST contain the following: (1) Sampling: Clinical samples (blood, urine, or wound swabs) are collected from patients suspected of having bacterial infections. (2) Isolation and Identification: Bacteria are isolated from the samples using standard microbiological techniques. Once isolated, the bacteria are identified using biochemical tests or automated systems, such as MicroScan identification panels [86–89]. (3) Preparation of Inoculum: A standardized inoculum is prepared, typically using a saline solution to match a specific optical density corresponding to 0.5 McFarland standard, which ensures consistent bacterial concentration across tests. (4) Inoculation of MicroScan Panels: The prepared inoculum is added to the MicroScan panels containing prefilled antibiotic dilutions. Each panel tests multiple antibiotics simultaneously [89–91]. (5) Incubation: The inoculated panels are incubated at 35 ± 1°C for 16–20 h in the MicroScan WalkAway system, which provides controlled conditions for bacterial growth. (6) Automated Reading: After incubation, the system automatically reads the panels to detect growth. The presence or absence of growth at specific antibiotic concentrations indicates susceptibility or resistance. (7) Interpretation of Results: Results are interpreted based on CLSI breakpoints, which categorize bacteria as susceptible, intermediate, or resistant to each tested antibiotic. This information is crucial for clinicians to select appropriate treatment regimens. (8) Reporting: Final results are generated and reported electronically, allowing healthcare providers to make timely decisions regarding patient management [89, 92, 93]. A study evaluated the use of MicroScan panels for the rapid identification and AST of Gram-negative rods isolated from positive blood cultures. In 1070 blood cultures, both conventional methods and direct identification/AST were performed concurrently, resulting in the determination of 9106 MICs. The direct method correctly identified approximately 96.5% of the isolates. The overall CA between the two methods was 92.86%. While the study identified 46 VMEs, the overall findings indicated a strong correlation with the standard method, particularly for *E. coli* and *K. pneumoniae*, with the exception of amoxicillin–clavulanate and piperacillin–tazobactam. For *Proteus mirabilis*, a good correlation was observed for beta-lactam antibiotics (excluding second- and third-generation cephalosporins). Additionally, *P. aeruginosa* showed good correlation for ciprofloxacin and gentamicin, and *Enterobacter cloacae* showed the same for amoxicillin–clavulanate, ciprofloxacin, gentamicin, and trimethoprim–sulfamethoxazole. A key benefit of this approach is that it delivers reliable results one day sooner than traditional methods, making it a straightforward, rapid, and cost-effective means of identifying pathogens and determining their antimicrobial susceptibilities from positive blood cultures [41]. In another investigation, a total of 207 isolates were tested, including three ATCC strains, one NCTC strain, six quality control strains from the Belgian National Antimicrobial Committee, and 197 clinical isolates comprising carbapenem-resistant Enterobacterales, *P. aeruginosa*, and *Acinetobacter baumannii*. Susceptibility categorization was based on the EUCAST breakpoints Version 13.1 from 2023. While overall CA and EA both exceeded 90%, several antibiotics crucial for treating multiresistant organisms exhibited CA and EA values below this threshold; specifically, meropenem, imipenem, and colistin for Enterobacterales, and meropenem and colistin for *P. aeruginosa*. The MicroScan NMDRM1 panel demonstrated a significantly higher resistance rate for meropenem, imipenem, amikacin, and colistin in Enterobacterales. The study's findings underscore the continued challenges in colistin susceptibility testing, emphasizing the critical need for the development of more precise commercial methods. Relying on a single commercial method may not ensure accurate MIC determination for colistin [43]. Ronat et al. assessed the performance of custom-designed MSF MicroScan microplates—the MICPOS1 for *Staphylococcus* and *Enterococcus* species, the MICNEG1 for Gram-negative bacilli, and the MICFAST1 for *Streptococcus* and *Haemophilus* species—using 387 isolates obtained from routine clinical bacteriology laboratories in low-resource settings. These were compared against reference methods. The study revealed that, for all antibiotics included on the three panels, the CA exceeded 90%, and the proportions of MEs and VMEs remained below 3%, in accordance with ISO standards. The implementation of the Prompt inoculation system was observed to elevate both the MIC values and the ME rate for certain antibiotics when testing *Staphylococcus* species. The accessibility of the manufacturer's user manual was deemed difficult for personnel with limited skills. However, the processes of inoculating and reading the panels were considered straightforward and easy to perform. In summary, the three MSF MicroScan MIC panels demonstrated satisfactory performance with clinical isolates from low-resource settings, offering a practical, reliable, and standardized AST method suitable for use in clinical bacteriology laboratories within these settings [85].

### 3.4. Sensititre (Thermo Fisher Scientific)

The Sensititre Automated Reading and Incubation System (ARIS) or Sensititre system, developed by Thermo Fisher Scientific, is an automated broth microdilution method used for testing antibiotic susceptibility. It allows for the determination of the MIC of various antibiotics against specific bacterial strains, facilitating rapid and accurate diagnosis of infections. The Sensititre system operates on the principle of broth microdilution, where bacteria are exposed to serial dilutions of antibiotics in a liquid medium. Each plate contains wells with different concentrations of antibiotics, allowing for simultaneous testing of multiple agents [94, 95]. The system uses fluorescence technology to detect bacterial growth and determine inhibitory endpoints, which enhances accuracy and reduces human error. The Sensititre Breakpoint Autoreader can provide results in as little as 5 h for certain tests, making it faster than traditional methods [96, 97]. The Sensititre system has demonstrated high sensitivity and specificity in various studies (Table 2). One study reported that the Sensititre dry-form broth microdilution MIC product showed a sensitivity of 99.5% and a specificity of 100% when testing ceftaroline against clinical isolates of *S. aureus*. These metrics indicate that the system is highly reliable for detecting antibiotic susceptibility. The Sensititre system is not primarily used for identifying bacteria; it focuses on AST for already identified bacterial strains. Typically, bacterial identification is performed using other methods, such as culture techniques or molecular methods (e.g., MALDI-TOF MS), before employing the Sensititre system to determine the susceptibility of the identified pathogens to various antibiotics [95, 107]. The steps involved in using Sensititre contain the following: (1) Sampling: Collect clinical samples (e.g., blood, urine, or tissue) suspected to contain pathogenic bacteria. Process the samples to isolate bacterial strains using standard microbiological techniques. (2) Preparation: Prepare a bacterial suspension by inoculating a suitable broth medium with the isolated bacteria to achieve a standardized concentration (typically 0.5 McFarland standard) [108–110]. (3) Loading the Plate: Dispense the prepared bacterial suspension into the wells of a Sensititre microdilution plate containing predefined concentrations of antibiotics. Ensure that each well is properly mixed to facilitate interaction between bacteria and antibiotics. (4) Incubation: Incubate the plate under appropriate conditions (usually at 35°C–37°C) for a specified duration (typically 18–24 h). (5) Reading Results: After incubation, use the Sensititre Breakpoint Autoreader to automatically read the results. The system detects fluorescence changes indicating bacterial growth or inhibition [108, 109, 111]. (6) Interpreting Results: Analyze the data generated by the reader to determine the MIC values for each antibiotic tested. Compare the MIC values against established breakpoints to classify bacterial susceptibility (susceptible, intermediate, or resistant). (7) Reporting: Compile and report results to healthcare providers, including recommendations for effective antibiotic therapy based on susceptibility patterns. The Sensititre system streamlines the process of AST, providing reliable results that aid in effective patient management and treatment decisions [108, 109, 111, 112]. The accuracy of the Sensititre Anaerobe MIC plate was evaluated using 56 clinically relevant anaerobic Gram-negative bacilli, including *Bacteroides fragilis* (*n* = 18), *Bacteroides thetaiotaomicron* (*n* = 10), *Bacteroides ovatus* (*n* = 4), *Bacteroides nordii* (*n* = 1), *Bacteroides uniformis* (*n* = 2), *Bacteroides buccae* (*n* = 1), and *Fusobacterium necrophorum* (*n* = 3). Anaerobic Gram-positive bacilli and cocci comprised 30% (*n* = 17) of the strains analyzed, specifically *Finegoldia magna* (*n* = 3), *Parvimonas micra* (*n* = 2), *Actinomyces oris* (*n* = 1), *Actinomyces meyeri* (*n* = 1), *Actinomyces neuii* (*n* = 2), *Propionibacterium avidum* (*n* = 2), *Cutibacterium* (*formerly Propionibacterium*) *acnes* (*n* = 2), and *Clostridium perfringens* (*n* = 4). Overall CA between the Sensititre Anaerobe MIC plate and the comparator method reached 95%. The Sensititre Anaerobe MIC plate demonstrated excellent accuracy for the majority of antibiotics tested. In instances where the Sensititre Anaerobe MIC plate and the ATB ANA test yielded discordant results, the gradient strip method was employed to resolve antimicrobial susceptibility categories. This resolution favored the Sensititre Anaerobe MIC plate in 58% of cases (21 of 36) for all antibiotics tested, with the exception of piperacillin, piperacillin–tazobactam, and penicillin. The gradient strip method indicated that the categorical differences observed for piperacillin, piperacillin–tazobactam, and penicillin were at least partially attributable to heterogeneity in resistance expression. Consequently, the Sensititre Anaerobe MIC plate presents a valuable alternative to the ATB ANA test for routine antimicrobial susceptibility testing of anaerobic bacteria in clinical microbiology laboratories [98]. A study was conducted to evaluate the performance of Sensititre EUMDROXF microplates, gradient strips, and disk diffusion for assessing imipenem/relebactam susceptibility in 148 clinical isolates of *P. aeruginosa*, using broth microdilution as a comparator and applying EUCAST 2024 breakpoints. The Sensititre microplates exhibited the highest accuracy (CA = 93.2%, EA = 93.9%, and a bias difference of +21.8%). The gradient strips demonstrated acceptable performance (CA = 84.5%; EA = 89.9%, and a bias difference of 21.0%), whereas the disk diffusion method misclassified 25.8% (16/62) of the resistant strains. These results emphasize the need for reliable testing methodologies, with Sensititre EUMDROXF microplates and gradient strips showing effectiveness in identifying imipenem/relebactam resistance in *P. aeruginosa* [99].

### 3.5. VersaTREK

The VersaTREK System is the only instrument that offers four FDA-cleared tests on one platform containing (1) blood culture, (2) sterile body fluids, (3) *Mycobacteria* and other bacteria detection, and *Mycobacterium tuberculosis* susceptibility testing, as well as providing efficiency, space savings, and cost containment. VersaTREK also is an automated device designed for testing antibiotic susceptibility, primarily used in microbiology laboratories. This system streamlines the process of determining the effectiveness of antibiotics against specific bacterial pathogens, enhancing efficiency and accuracy in clinical diagnostics [29, 102, 113]. The device can assist in identifying bacterial species based on their growth patterns and metabolic activity. The VersaTREK system operates on the principle of real-time monitoring of microbial growth. It utilizes a colorimetric method to detect changes in pH that occur as bacteria metabolize nutrients in the medium. VersaTREK is the only system capable of detecting any gas produced or consumed by organisms. Because it is not limited to CO_2_ production, like other systems, VersaTREK is able to detect a wider range of both common and fastidious organisms [103, 113–115]. This unique detection technology means faster results with fewer limitations, reducing length of stay and therapy costs, and promoting better patient care. This allows for the assessment of bacterial growth in the presence of various antibiotics, indicating susceptibility or resistance. The system measures changes in the medium's color, which correlates with bacterial growth based on detection of metabolic activity. It automatically analyzes the data to determine the MIC for each antibiotic tested, providing rapid results [47, 113, 115]. Just two bottles are all you need to recover organisms from adults, pediatrics, and patients on antibiotics, reducing media costs and simplifying inventory control. VersaTREK REDOX media have many distinct features, including (1) only media FDA-cleared for draws as low as 0.1 mL without additional supplements, perfect for pediatric patients, (2) largest dilution ratio in the industry (1:9), allowing dilution of serum host factors, and (3) only FDA-cleared true direct-draw bottle on the market, no need for costly blood collection adapters [47, 116]. The steps involved in using VersaTREK for AST include the following: (1) Sampling: Collect a clinical specimen (e.g., blood, urine, or swab) from the patient suspected of having an infection. (2) Inoculation: Introduce the specimen into the VersaTREK culture bottles containing a suitable growth medium. (3) Antibiotic Addition: Add various concentrations of antibiotics to different bottles to test their efficacy against the isolated bacteria. (4) Incubation: Place the inoculated bottles into the VersaTREK system, where they are incubated under controlled conditions [47, 113]. (5) Monitoring Growth: The system continuously monitors bacterial growth by measuring changes in pH and color in real-time. (6) Data Interpretation: Once incubation is complete, analyze the results displayed by VersaTREK to determine which antibiotics inhibited bacterial growth effectively. (7) Reporting Results: Generate a report that includes MIC values for each antibiotic tested, guiding clinicians in selecting appropriate treatment options. By following these steps, laboratories can efficiently determine antibiotic susceptibility, ultimately aiding in effective patient management and treatment decisions [47, 113]. A clinical evaluation of the VersaTREK 528 Blood Culture System was conducted in a Chinese tertiary hospital. Confirmed bacterial species (*n* = 78), including 43 Gram-positive isolates, 30 Gram-negative isolates, and 5 *Candida albicans* strains, were individually inoculated into blood culture bottles. In a simulated blood culture experiment, 90% (63/70) of the VersaTREK aerobic bottles yielded positive results, which was a higher rate than that observed with the FX 400 system (59/70, 84%). In clinical blood cultures, the VersaTREK BC system, when inoculated with either 5 or 10 mL of patient blood, exhibited performance comparable to the FX system using 10 mL of patient blood [102]. Another study focused on validating a novel diagnostic strategy that combines the VersaTREK system for bacterial recovery with real-time PCR for the identification of *Mycobacterium chimaera* in water samples. The VersaTREK system detected an initial bacterial load of 100 CFU in less than 3 days. The detection limit did not appear to be affected by NaOH decontamination or the initial volume of the water sample. Analytical sensitivity was determined to be 1.5 × 10^2^ CFU/mL, and positivity was generally detected within 15 days. VersaTREK has the potential to accelerate mycobacterial growth in culture. When used in conjunction with PCR, it can improve the overall recovery of mycobacteria from environmental samples. This combined approach could be valuable for microbial control in hospital settings, as well as in environments with low-level contamination by viable mycobacteria [103].

### 3.6. PREVI Isola System

PREVI Isola is an automated system designed for microbiology laboratories. It streamlines the process of inoculating culture media with urine samples, enhancing efficiency and accuracy in diagnosing urinary tract infections (UTIs). The development of PREVI Isola is part of a broader trend toward automation in laboratory diagnostics. Automated systems, such as PREVI Isola, have emerged to address the increasing demand for rapid and reliable microbiological testing, particularly in high-throughput environments, such as hospitals. While specific historical details regarding the inception of PREVI Isola are sparse, its introduction reflects advancements in laboratory technology aimed at improving patient care through more efficient diagnostic processes. This system detects bacterial growth and determines antibiotic susceptibility. The PREVI Isola system is not only utilized for urine samples but also plays a significant role in processing wound, body fluid, stool, genital, and blood samples in microbiology laboratories [104, 117, 118]. The system automates the tedious and repetitive task of inoculating culture plates, allowing laboratory technicians to focus on more complex tasks. This automation standardizes the inoculation process, reducing variability and improving reproducibility across samples [119]. PREVI Isola can handle up to 180 plates per hour, significantly increasing laboratory productivity. The system is capable of processing various types of samples, including blood cultures, urine, and body fluids. Each inoculated plate is tagged with a barcode that contains essential patient and specimen information, enhancing traceability. The standardized inoculation process also aids in meeting accreditation requirements for laboratories [119]. The system has shown high concordance rates with manual methods in terms of both qualitative and quantitative aspects of culture results. For instance, studies report concordance rates of 100% for blood cultures, indicating its reliability [104, 119]. While PREVI Isola itself does not directly identify bacteria, it can be linked to automated systems that perform microbial identification, such as mass spectrometry (e.g., MALDI-TOF) or biochemical tests. These systems analyze isolated colonies to provide specific bacterial identification based on their biochemical profiles or mass spectrometric data [54]. PREVI Isola automates the inoculation of cultured plates and plays a role in AST. This is done to grow sufficient bacterial colonies for testing. After the bacterial growth is established on the agar plates, PREVI Isola automatically places antibiotic disks onto the surface of the agar. This step is crucial as it allows for the assessment of how effectively each antibiotic can inhibit bacterial growth. The inoculated plates with antibiotic disks are then incubated under controlled conditions to allow for bacterial growth and interaction with the antibiotics. After incubation, the system measures the zones of inhibition around each antibiotic disk [120]. The PREVI Isola uses a patented applicator to inoculate agar plates with the bacterial suspension. The system employs a new protocol that involves eight calibrated deposits of the bacterial inoculum and two rounds of rotation to ensure even distribution across the agar surface. This automated inoculation process allows for rapid seeding of up to 180 plates per hour, significantly improving efficiency compared to manual methods, which can take longer. After inoculation, antibiotic-impregnated disks are placed on the agar surface. The PREVI Isola can automate this step as well, ensuring precise placement and spacing of the disks to prevent overlap. The size of these zones indicates the susceptibility or resistance of the bacteria to specific antibiotics [120]. The steps involved in performing the PREVI Isola technique contain the following: (1) Sampling: Collect clinical samples using sterile containers to prevent contamination. (2) Preparation: Prepare culture media according to standard protocols. (3) Inoculation Using PREVI Isola: Place samples into the PREVI Isola system. The system automatically streaks the sample onto the prepared culture plates in a standardized manner. (4) Incubation: Incubate the inoculated plates at specified temperatures (usually 35°C–37 °C) for 24 h to allow bacterial growth. (5) Observation and Counting: After incubation, visually inspect each segment of the plate. Count colonies and assess their morphology to identify bacterial species [48, 104, 120]. (6) Quantification: Use the segmented growth pattern to quantify CFUs, noting which segments correspond to different CFU thresholds (e.g., < 10^3^, 10^4^, ≥ 10^5^ CFU/mL). (7) AST: Perform further tests (e.g., disk diffusion or broth microdilution) on isolated colonies to determine antibiotic susceptibility profiles. (8) Interpreting Results: Analyze data based on established guidelines (e.g., CLSI criteria) to classify bacteria as susceptible, intermediate, or resistant to specific antibiotics. Report findings to clinicians for appropriate treatment decisions [48, 54, 104, 120]. Choi et al. assessed the performance of the PREVI Isola automated inoculation system for processing various routine clinical samples, including blood. A total of 377 nonduplicate samples (102 blood cultures, 203 urine samples, and 72 body fluid samples) were collected and inoculated. The concordance rate for quality was 100% for blood cultures, 97.0% for urine samples, and 98.6% for other body fluids. In terms of quantity, the concordance rates were 98.0%, 97.0%, and 95.8%, respectively. While the PREVI Isola required slightly more time to inoculate specimens compared to the manual method, the hands-on time was significantly reduced. The decrease in hands-on time with the PREVI Isola was approximately 6 min per 10 samples. The PREVI Isola demonstrated high concordance with the manual method in the inoculation of various body fluids, particularly blood culture samples. The implementation of PREVI Isola in clinical microbiology laboratories is anticipated to result in substantial time and human resource savings [104]. Fecal and genital swab samples from 100 different patients were processed both manually and using the PREVI Isola system. All samples were suitable for evaluation. No *Salmonella* or *Shigella* species were detected in either group. *Campylobacter* species were identified in 5 samples, but the PREVI Isola method allowed for better distinction of individual, suspected colonies, which were also observed earlier (after 1 day). High counts of *Yersinia* species were detected in a single sample, but only with the PREVI Isola method. For genital swab results, the counts of various bacteria were generally somewhat higher (+) with the PREVI Isola method compared to manual inoculation. With PREVI Isola, individual colonies of the different bacteria were much easier to distinguish. No difference was observed in the isolation of *Neisseria gonorrhoeae* (3 samples) between the two methods. *Gardnerella* species were observed one day earlier and were much easier to distinguish from other bacteria using the PREVI Isola method. As with urine samples, PREVI Isola led to more easily readable results for the more challenging cultures of fecal and genital swabs. Individual suspected colonies were more readily distinguished, and bacterial counts were higher. Suspected colonies were also often positive one day earlier. Therefore, PREVI Isola is a valuable tool in the time-consuming culture of both fecal and, especially, genital swab samples, enabling earlier identification of organisms [48]. A total of 350 bronchopulmonary specimens were collected from a university-affiliated hospital and processed in parallel using both a manual reference method and an automated method. The specimens included expectorations (*n* = 75), bronchoalveolar lavages (*n* = 68), tracheal aspirations (*n* = 17), and protected distal samples (*n* = 190). The automated method incorporated a specific enumeration reading grid, a preliquefaction step, and a fluidity test performed before inoculation. The qualitative concordance (i.e., the agreement in the number of specimens exceeding the clinical threshold for bacterial count) was 100%, and the quantitative concordance (i.e., the agreement within a 0.5 log value) was 98.2%. The implementation of an automated decapper would further enhance the biosafety of the process. The adapted procedure using the PREVI Isola offers a time- and cost-effective method for processing bronchopulmonary specimens [105].

### 3.7. Mindray TDR System

In recent years, the market has seen the emergence of various innovative automated systems. One notable example is the Mindray TDR system, which originates from Shenzhen Mindray Bio-Medical Electronics in China. This system provides blood culture bottles designed for both aerobic and anaerobic applications across different age groups [49]. The TDR microorganism analysis system offers 10 categories of test cards, covering a wide range of microorganisms including *Enterobacteriaceae, Micrococcaceae, Streptococcus*, yeast-like fungi, *Vibrionaceae*, nonfermentative bacteria, *Bacillus*, Coryneform bacteria, *Neisseria*, *Haemophilus*, and other fastidious bacteria, such as anaerobes. Identification and antimicrobial susceptibility testing are performed using colorimetry and turbidimetry methods, respectively [121, 122]. The Mindray TDR system operates by tracking changes in carbon dioxide levels generated by microbial activity within blood culture bottles. It utilizes a colorimetric approach to quantify these changes. Given its recent introduction to the field of blood culture diagnostics, comprehensive studies on its performance are scarce. When microorganisms are present in a sample, they break down nutrients from the medium, resulting in increased CO_2_ output. As CO_2_ concentrations increase, sensors positioned at the base of each bottle undergo a color transformation from blue–green or grayish tones to yellow. This visual cue can be manually observed or automatically detected by the system to signal microbial growth [49]. The steps involved in performing the Mindray TDR system contain the following: (1) Sampling Collection: Obtain clinical blood samples from patients using sterile techniques to prevent contamination. (2) Sample Preparation: Immediately after inoculation, the vials were simultaneously loaded into blood culture devices and incubated. (3) Incubation: Incubation periods of blood culture devices were set to five days (120 h). At the end of the five-day incubation period, the bottles that did not give a growth signal were considered negative, and the incubation was terminated. All vials giving positive signals were inoculated onto sheep blood agar and incubated at 37°C for 16–24 h. (4) Analysis Using Automated Systems: Use colorimetry to detect changes in color due to metabolic activities of microorganisms on different substrates. For AST, prepare panels containing antibiotics at varying concentrations. Inoculate these panels with the isolated bacteria and incubate them under controlled conditions. Measure changes in optical density (turbidity) over time as an indicator of bacterial growth inhibition by antibiotics [49, 106, 123]. (5) Interpretation of Results: The system analyzes color changes across different substrates and matches them against a database to identify the organism based on its biochemical profile. The system interprets turbidity measurements over time for each antibiotic concentration tested. It determines MIC, which are used to classify bacteria as susceptible or resistant based on established breakpoints [49, 106, 123]. A comparative study assessed the performance of TDR-300B and VITEK 2 in identifying *P. aeruginosa* against the benchmark of VITEK-MS results. Each isolate was tested by inoculating a single colony into both a TDR-300B NF-64 card and a VITEK 2 GN cassette after confirming they were oxidase-positive Gram-negative rods. Compared to VITEK-MS, TDR-300B showed an agreement rate of approximately 80%, while VITEK 2 achieved about 92%. Both systems demonstrated high sensitivity—95% for TDR-300B and perfect sensitivity for VITEK 2—though TDR-300B had lower predictive value accuracy compared to VITEK, albeit not significantly so in statistical terms [106]. The time-to-detection performance of the Mindray TDR and the BacT/ALERT3D blood culture systems was compared using simulated blood cultures. A total of 352 blood culture bottles were evaluated (176 with BacT/ALERT3D and 176 with Mindray TDR-X060), consisting of 336 aerobic and 16 anaerobic bottles. At both 10 and 100 CFU/mL dilutions, no significant difference was observed between the two systems in terms of mean detection times across all isolates (*p*=0.965, *p*=0.245). When stratified by organism type, the detection time for Gram-positive bacteria at the 10 CFU/mL dilution was significantly shorter in the BacT/ALERT system (*p*=0.019), whereas the Mindray system exhibited a significantly shorter detection time for yeasts (*p*=0.047). The sample size of anaerobic bacteria was insufficient for drawing statistical conclusions; however, a trend toward shorter detection times was noted with the Mindray TDR-X060 system. These two systems, which operate on similar principles, demonstrated different concentration-dependent performances regarding positivity detection times depending on the type of microorganism. While the Mindray TDR-X060 system has been deemed safe for use at higher concentrations, further comparative studies are warranted at lower concentrations to fully evaluate the newly introduced Mindray system [49].

## 4. Discussion and Outlook for Next Generation of Automated AST

Automated AST systems represent a significant advancement over traditional manual methods, offering improved turnaround times, accuracy, and standardization. Nonetheless, each modality presents specific limitations that must be recognized to optimize their clinical utility [124, 125]. VITEK 2 utilizes automated broth microdilution with expert system software, providing rapid and reliable results, particularly for common bacterial pathogens. However, discrepancies have been reported for certain antimicrobials—such as amoxicillin–clavulanate and meropenem—especially when testing challenging organisms, such as Staphylococci, implying that further refinements are required to reduce VME rates [36, 124, 126]. The BD Phoenix system, based on oxidation–reduction indicators and biochemical reactions, offers high concordance with traditional methods but shows limitations in detecting carbapenemase production accurately, with high sensitivity but low specificity in some cases, necessitating confirmatory tests for critical resistance determinants. Additionally, false susceptibility reports for colistin-resistant strains highlight the need for complementary testing methods [127–129]. MicroScan effectively performs broth microdilution susceptibility testing with good agreement for many pathogens but encounters reduced accuracy with some antibiotics, including colistin and carbapenems, especially for multidrug-resistant Gram-negative bacteria. Also, performance in low-resource settings has been promising, yet challenges remain concerning accessibility and training [43, 130, 131]. The Sensititre system demonstrates high sensitivity and specificity, particularly for anaerobic bacteria, using broth microdilution and fluorescence detection. Despite its strengths, some discrepancies with gradient strip methods suggest that heterogeneity in resistance expression may affect CA, especially for beta-lactam antibiotics [43, 95, 132]. VersaTREK offers a unique detection modality via pressure changes reflecting microbial metabolism and gas detection, capable of handling diverse organisms, including slow-growing mycobacteria. However, the longer incubation times required for slow grower detection limit its utility for urgent clinical decision-making. While it shows promise when combined with molecular techniques, cost and workflow considerations may restrict its wide adoption [133, 134]. The PREVI Isola system automates specimen inoculation and antibiotic disk placement, improving standardization and reducing hands-on time. Nonetheless, it performs culture-based AST, thereby maintaining the inherent delays associated with culture growth. Its primary benefit lies in facilitating earlier and more reproducible growth detection rather than accelerating susceptibility results [54, 104]. The Mindray TDR system—with integrated MIC detection through colorimetry and turbidimetry—is a newer entrant with promising results comparable to established systems. However, limited data on its performance across a broader spectrum of pathogens and resistance mechanisms warrant further validation before routine clinical implementation [39, 49, 106]. Future advancements in automated AST should focus on integrating rapid phenotypic testing with molecular diagnostics to overcome current limitations, such as slow growth detection and resistance mechanism diversity. Emerging microfluidic platforms and machine learning-enhanced systems hold the potential to deliver susceptibility results within hours directly from clinical specimens, significantly impacting patient management [135, 136]. Artificial intelligence (AI) integration can provide real-time data analysis to streamline workflows, reduce errors, and automate negative culture result reporting without human oversight—a key step toward fully autonomous clinical microbiology laboratories. Moreover, improvements in sensitivity for slow-growing bacteria, enhanced detection of mixed infections, and expansion of resistance marker panels will be crucial. Cost-effectiveness and adaptability to low-resource settings remain priorities to ensure global accessibility [137–139].

## 5. Conclusion

Clinical microbiology laboratories have seen significant advancements with automated systems capable of handling the complex and varied procedures involved in culture-based AST. Some research has highlighted the benefits of these systems, impacting multiple aspects including patient care, efficiency, tracking, and quality assurance practices. Recent developments in full automation have reinforced these benefits. However, there is a need to further establish the clinical effectiveness of these technologies. To date, research on how rapid microbiological methods impact patient outcomes, healthcare costs, and antibiotic usage is limited, with studies yielding mixed yet promising results. The integration of AI into bacterial detection and AST is poised to streamline workflow, ensuring consistent performance. AI to automatically process negative cultures and release results without human oversight is a pressing requirement. Moving ahead, it will be crucial to assess the cost-effectiveness and clinical value of these innovative microbiology tests.

## Figures and Tables

**Table 1:** Comparison of automated AST systems and their operational principles.

AST system	Principle	Detection time	Reference
VITEK 2 (bioMérieux)	Automated broth microdilution with 64-well cards; uses advanced expert system for resistance detection	4–18 h	[4, 39]
Phoenix (BD)	Oxidation–reduction indicator detecting bacterial growth in the presence of antimicrobials	Up to 18 h (read every 20 min)	[4, 40]
MicroScan (Beckman Coulter)	Automated broth microdilution panels with RENOK inoculator	4.5–18 h	[4, 41, 42]
Sensititre (Thermo Fisher Scientific)	Broth microdilution in 96-well microplates measuring turbidity	∼18–24 h	[43, 44]
VersaTREK	Automated microbial growth detection via pressure changes from oxygen consumption	24–48 h	[45, 46]
PREVI Isola system	Automated specimen processing and streaking for culture prep	Culture prep time only	[47, 48]
Mindray TDR system	Automated broth microdilution with integrated MIC detection	∼16–20 h	[39, 49]

**Table 2:** An overview of clinical studies that use automated systems to examine AST and identify microorganisms.

Name of automated AST	Year	Sample	Name of infection	Results	Reference
VITEK 2 (bioMérieux)	2023	Blood	Gram-negative rods or gram-positive cocci	The overall categorical and essential agreement (EA) rates were notably high at 98.4% and 96.7%, respectively. Overall, direct AST from positive blood culture broths using VITEK 2 proved reliable and efficient for Enterobacterales and *Staphylococci*, potentially enhancing antimicrobial stewardship.	[36]
	2022	Urinary tract infections and pyelonephritis	Enterobacterales and *Pseudomonas aeruginosa*	These positive findings supporting VITEK's accuracy in automated susceptibility testing compared against manual methods, such as broth microdilution tests, are used traditionally as benchmarks in clinical microbiology laboratories worldwide today.	[38]
	2024	Hospital wastewater	*Salmonella typhimurium, Salmonella paratyphi, Salmonella enteritidis, Salmonella enterica*, and *Salmonella typhi*	Of the total of 136 isolates analyzed at the species level, an impressive accuracy rate of approximately 98.5% was achieved in identifying them correctly as belonging to specific species—134 out of every possible case being accurately classified. Overall EA was calculated at approximately 98.86%, with a categorical agreement rate around 95.95%.	[67]

Phoenix (BD)	2006	Clinical isolates recovered from routine cultures in the clinical microbiology laboratory	Staphylococci and enterococci	The overall concordance rates for genus and species identification were 99.7% and 99.3%, respectively. Both *Staphylococcus aureus* and enterococci were accurately identified using the Phoenix system. Overall, the Phoenix system performed comparably to traditional methods for identifying and conducting AST on these microorganisms.	[37]
	2018	Clinical isolates	*Enterobacteriaceae*	Both the BD Phoenix system and the Rapid Polymyxin NP test successfully identified the 16 isolates of *Escherichia coli, Klebsiella pneumoniae*, and *S. enterica* that carried plasmid-encoded *mcr*-1 and *mcr*-2 genes.	[82]
	2021	Blood cultures, rectal swabs, urine, and respiratory material	Enterobacterales and nonfermentative Gram-negative rods	The performance assessment revealed that the PCD assay demonstrated an overall sensitivity of 98.29% but a specificity of 17.95% for detecting carbapenemase production.	[80]
	2024	Repository samples	*Streptococcus pneumoniae*	The results showed that all methods achieved an EA of at least 90% when compared to broth microdilution.	[81]

MicroScan (Beckman Coulter)	2021	Blood	Gram-negative rods	The direct method correctly identified approximately 96.5% of the isolates. The overall categorical agreement between the two methods was 92.86%.	[41]
	2024	Blood cultures, sputum, abdominal samples, and urine	Enterobacterales, *P. aeruginosa*, and *Acinetobacter baumannii*.	While overall CA and EA both exceeded 90%, several antibiotics crucial for treating multiresistant organisms exhibited CA and EA values below this threshold; specifically, meropenem, imipenem, and colistin for Enterobacterales, and meropenem and colistin for *P. aeruginosa*.	[43]
	2022	Bloodstream pathogens or contaminants	*Staphylococcus, Enterococcus,* Gram-negative bacilli, *Streptococcus*, and *Haemophilus*	The study revealed that, for all antibiotics included on the three panels, the category agreement exceeded 90%. The three MSF MicroScan MIC panels demonstrated satisfactory performance with clinical isolates from low-resource settings, offering a practical, reliable, and standardized AST method suitable for use in clinical bacteriology laboratories within these settings.	[85]

Sensititre (Thermo Fisher Scientific)	2018	Blood cultures, peritoneal fluids, tissue biopsies, bronchoalveolar lavage, and pus samples	Anaerobic Gram-negative bacilli, anaerobic Gram-positive bacilli and cocci	Overall categorical agreement between the Sensititre™ Anaerobe MIC plate and the comparator method reached 95%. The Sensititre™ Anaerobe MIC plate presents a valuable alternative to the ATB ANA® test for routine antimicrobial susceptibility testing of anaerobic bacteria in clinical microbiology laboratories.	[98]
	2024	Clinical strains were isolated from various infection sites	*P. aeruginosa*	The Sensititre microplates exhibited the highest accuracy (CA = 93.2%, EA = 93.9%, and a bias difference of +21.8%). These results emphasize the need for reliable testing methodologies, with Sensititre EUMDROXF® microplates and gradient strips showing effectiveness in identifying imipenem/relebactam resistance in *P. aeruginosa*.	[99]

MALDI-TOF MS	2018	Blood	*E. coli*	MBT-ASTRA correctly identified 95% of the AMX-susceptible isolates and 84% of the CTX-susceptible isolates. At the initial report of a positive blood culture, MALDI-TOF MS could provide prescribers with bacterial identification and AMX/CTX susceptibility results, potentially reducing the use of broad-spectrum antibiotics.	[100]
	2021	Clinical strains	*K. pneumoniae*	The agreement between MIC values obtained from MALDI-TOF MS analysis and those from the broth microdilution method was 61.7% for ceftriaxone and 71.7% for imipenem. Applying the CLSI breakpoints for ceftriaxone and imipenem resistance, the 60 isolates were accurately classified as resistant or susceptible, exhibiting 100% sensitivity and 100% specificity.	[101]

VersaTREK	2018	Blood	Gram-positive isolates, Gram-negative isolates, and *Candida albicans* strains	In a simulated blood culture experiment, 90% (63/70) of the VersaTREK aerobic bottles yielded positive results, which was a higher rate than that observed with the FX 400 system (59/70, 84%). In clinical blood cultures, the VersaTREK BC system, when inoculated with either 5 or 10 mL of patient blood, exhibited performance comparable to the FX system using 10 mL of patient blood.	[102]
	2021	Water samples	*Mycobacterium chimaera*	The VersaTREK™ system detected an initial bacterial load of 100 CFU in less than 3 days. This combined approach could be valuable for microbial control in hospital settings, as well as in environments with low-level contamination by viable mycobacteria.	[103]

PREVI Isola system	2018	Blood cultures, urine samples, and body fluid samples	*E. coli, P. aeruginosa, A. baumannii, Enterobacter cloacae, K. pneumonia, Proteus mirabilis, Pseudomonas stutzeri, Aeromonas* spp.*, Citrobacter freundii, Stenotrophomonas maltophilia, Staphylococcus epidermidis, S. aureus, Staphylococcus capitis, Enterococcus faecium, Corynebacterium jeikeium, Enterococcus avium, Enterococcus faecalis, Staphylococcus mitis, Corynebacterium striatum, Staphylococcus haemolyticus, Staphylococcus hominis, Staphylococcus pyogenes,* and *Staphylococcus warneri*	The concordance rate for quality was 100% for blood cultures, 97.0% for urine samples, and 98.6% for other body fluids. The decrease in hands-on time with the PREVI Isola was approximately 6 min per 10 samples. The implementation of PREVI Isola in clinical microbiology laboratories is anticipated to result in substantial time and human resource savings.	[104]
	2010	Fecal and genital swab samples	*Salmonella, Shigella, Campylobacter, Yersinia, Neisseria gonorrhoeae, and Gardnerella*	With PREVI Isola, individual colonies of the different bacteria were much easier to distinguish. As with urine samples, PREVI Isola led to more easily readable results for the more challenging cultures of fecal and genital swabs. Individual suspected colonies were more readily distinguished, and bacterial counts were higher. Suspected colonies were also often positive one day earlier. Therefore, PREVI Isola is a valuable tool in the time-consuming culture of both fecal and, especially, genital swab samples, enabling earlier identification of organisms.	[48]
	2013	Expectorations, bronchoalveolar lavages, tracheal aspirations, and protected distal samples	*Haemophilus influenzae, S. pneumoniae,* and Enterobacteria	The qualitative concordance (i.e., the agreement in the number of specimens exceeding the clinical threshold for bacterial count) was 100%, and the quantitative concordance (i.e., the agreement within a 0.5 log value) was 98.2%. The adapted procedure using the PREVI Isola offers a time- and cost-effective method for processing bronchopulmonary specimens.	[105]

Mindray TDR system	2024	Sputum samples along with nonsputum samples, including those from the appendix, blood, feces, eye scab, pus, throat, and urine.	*P. aeruginosa*	TDR-300B showed an agreement rate of approximately 80% and demonstrated high sensitivity—95% for TDR-300B and though TDR-300B had lower predictive value accuracy compared to VITEK, albeit not significantly so in statistical terms.	[106]
	2021	Blood	*K. pneumonia, E. cloaca, Enterobacter hormaechei, E. coli, P. mirabilis, S. aureus, S. epidermidis, P. aeruginosa, A. baumannii, Streptococcus mutans, S. pyogenes, C. albicans—parapsilosis, E. faecalis,* and *Enterococcus casseliflavus*	The system demonstrated different concentration-dependent performances regarding positivity detection times depending on the type of microorganism. While the Mindray TDR-X060 system has been deemed safe for use at higher concentrations, further comparative studies are warranted at lower concentrations to fully evaluate the newly introduced Mindray system.	[49]

## Data Availability

All data supporting the findings of this study are included within the article. No additional or supporting information are available. Other researchers may access and utilize the information directly from the published content.
